# Effect of First Long-Term Training on Whole Blood Count and Blood Clotting Parameters in Thoroughbreds

**DOI:** 10.3390/ani11020447

**Published:** 2021-02-09

**Authors:** Arianna Miglio, Emanuela Falcinelli, Anna Maria Mezzasoma, Katia Cappelli, Samanta Mecocci, Paolo Gresele, Maria Teresa Antognoni

**Affiliations:** 1 Department of Veterinary Medicine, University of Perugia, Via San Costanzo 4, 06126 Perugia, Italy; katia.cappelli@unipg.it (K.C.); samanta.mecocci@studenti.unipg.it (S.M.); maria.antognoni@unipg.it (M.T.A.); 2 Department of Medicine and Surgery, Section of Internal and Cardiovascular Medicine, University of Perugia, Via San Costanzo 4, 06126 Perugia, Italy; annamezzasoma@gmail.com (A.M.M.); paolo.gresele@unipg.it (P.G.)

**Keywords:** whole blood count, blood clotting, hypercoagulability thrombin-antithrombin complex, equine, racehorse training, Thoroughbreds

## Abstract

**Simple Summary:**

Sport training is known to induce changes in blood parameters due to the acute physical effort. However, only a few studies have been carried out on the effects of long-term exercise on blood parameters. The Thoroughbred racehorse is a valid animal model to investigate such changes. Twenty-nine clinically healthy 2-year-old Thoroughbred racehorses were followed during their first 4 months of sprint training. Blood was collected at rest, once a month, five times during the sprint training period. In each sample, complete blood count, clotting factors and thrombin–antithrombin complexes were measured. The sprint training period induced significant modifications over time of numerous hematological and clotting parameters compared to pre-training levels. The first long-term training induces fundamental hematological and blood clotting changes in untrained Thoroughbreds, most likely as a result of the physiologic adaptation to training.

**Abstract:**

Training has a strong effect on the physiology of hematological parameters and blood coagulation, both in humans and in horses. Several blood changes have been reported after exercise in horses but available data differ. We aimed to investigate modifications in complete blood count and some hemostatic parameters induced by the first training period in young untrained Thoroughbred racehorses to detect a possible labile blood coagulability in racehorses. Twenty-nine untrained 2-year-old Thoroughbreds were followed during their incremental 4-month sprint exercise schedule. Blood collection was performed once a month, five times (T-30, T0, T30, T60 and T90), before and during the training period for measurement of complete blood count (CBC) and blood clotting parameters (prothrombin time—PT, activated partial prothrombin time—APTT, thrombin clotting time—TCT, fibrinogen—Fb, thrombin–antithrombin complex—TAT). Differences among the time points for each parameter were analyzed (ANOVA, Kruskal–Wallis one-way analysis of variance, *p* < 0.05). In Thoroughbreds, the first long-term exercise workout period was found to induce a statistical increase in red blood cell indexes and lymphocytes, eosinophils and platelet counts, as well as a hypercoagulability state evident at 30 days of training, which returned to basal levels after 90 days. Regular physical exercise seems to blunt the negative effects of acute efforts on hematological and clotting parameters, an effect that may be attributed to the training condition.

## 1. Introduction

During physical exercise, both in humans and in the athletic horse, blood adaptations occur to guarantee adequate transport of oxygen and blood-borne substrates to muscles as well as removing metabolites. The principal method to assess the effect and efficacy of training is to verify and monitor the modifications of blood constituents relative to the duration, type, frequency and intensity of workloads in the different athletic disciplines [1,2,3,4]. 

The most known physiological hematological changes correlated with acute and intense training in sport horses are an increase in the hematocrit (HCT), red blood cells (RBC) and hemoglobin (Hb) values that tend to return to basal levels after exercise. Moreover, an increase in platelet (Plt) count that does not necessarily return immediately to the rest levels and a slight increase in white blood cells (WBC) are demonstrated. In particular, WBC show a decrease in the neutrophils to lymphocyte ratio in association with an increase in the lymphocyte number [2,5,6]. No equine studies have been carried out on the effect of long-term training on hematological parameters, with the exception of one study evaluating the white blood cell count throughout a long timeframe in Thoroughbreds [3]. More studies are needed to improve the knowledge of the modifications of these parameters in racehorses after the first intense workload. 

Training has a large effect on the physiology of blood coagulation both in humans and in horses [7,8,9,10,11,12,13]. Available data reporting hemostatic changes after exercise in horses are relatively scarce and not unequivocal. Generally, as happens in humans, the modifications of clotting parameters seem to indicate a more labile blood coagulability in athletic horses, possibly due to possible microvascular trauma induced by exercise [7]. In particular, in equine medicine, published data on the effects of exercise on clotting factors differ and are ambiguous. Some researchers found that exercise increased the coagulation time in healthy horses, indicative of a hypocoagulative status [14], whereas others found a decreased clotting time to be indicative of a hypercoagulative status [7,15,16] or an unchanged coagulative status [10]. Findings in this field are equivocal, substantially because they are related to different types of training schedules (different intensity, duration and type of exercise), different races of horse, different performance statuses of animals (trained or untrained horses) and different methods used to perform analysis. Moreover, in the last few years, the majority of studies have been carried out mostly to evaluate the immediate effect of exercise [11,15,16,17,18,19,20], reporting that exercise affects the hemostatic process associated with an acute and transient increase in blood coagulability that seems to be counterbalanced by a simultaneous increase in fibrinolytic activity depending on both the intensity and duration of exercise [10,21]. Conversely, only a few studies have been carried out on the long-term effects of training on the hemostatic system in racehorses and they focused on the changes in clotting times [7,10,13] and platelets aggregation [9,13]. They supposed that the modifications of the clotting mechanism in response to a long training period could be considered as a normal physiological response of the hemostatic system to exercise; however, they showed different results, and as such, more studies are needed to increase the knowledge on this topic in sports horses. 

The thrombin–antithrombin complex (TAT) is considered to be an effective marker that can detect prothrombotic status earlier than other laboratory tests. Determination of TAT levels is considered useful in identifying coagulation changes in human patients with deep vein thrombosis and/or pulmonary thromboembolism [22,23,24,25]. Antithrombin, the most abundant natural anticoagulant, regulates coagulation by forming the inhibitory TAT complex. In veterinary medicine, using TAT remains uncommon and there is a limited number of reports demonstrating TAT as an effective coagulation marker in animals. However, some reports suggest TAT is valid in assessing prothrombotic status in dogs (Cushing’s syndrome, malignant tumors and CID) [25,26,27]. Nevertheless, data about TAT complex levels in athletic horses are rare [16,28] and no studies have been carried out in racehorses. This lack of evidence suggests more studies are needed to investigate if it may hold the promise of being an indicator of increased thromboembolic risk also in racehorses where exercise-induced pulmonary hemorrhages (EIPH) are a severe problem.

Therefore, the aim of the present study was to investigate the modifications of complete blood count, clotting parameters and the TAT complex in young, untrained Thoroughbred racehorses in the first 4-month timeframe of incremental training in order to deeply understand the dynamics of the hemostatic system in racehorses. Monitoring coagulation factors in these animals could provide useful information about their fitness level during exercise training and competitions. Moreover, it could also help in developing advanced training programs and lead to a better understanding and prevention of hemorrhagic syndromes in equine athletes.

## 2. Materials and Methods

### 2.1. Ethical Animal Research

Blood sampling was approved by the Italian Horse Racing Board and performed by the authorized veterinary practitioner during routine controls to assess the health of the animals in the course of the training season. Prior to sample collection, written owner or trainer consent was obtained for each animal. The animal care procedures were compliant with the European recommendations (Directive 2010/63/EU) for the protection of animals used for scientific purposes.

### 2.2. Animals

Twenty-nine Thoroughbred horses (17 males and 12 females; 2 years old) were included in this study. Horses were considered to be clinically healthy if they did not have a history of hemostatic abnormalities and, at each sample collection, heart exam (cardiac auscultation, assessment of quality and regularity of the peripheral pulse by palpation of the facial artery), respiratory auscultation, rectal temperature, routine hematology and serum biochemistry analyses were within normal limits [29,30]. No pharmacological treatment was administered before the study.

All horses were managed similarly (individual housing, a natural photoperiod, natural indoor temperature and the same feeding schedule). These animals were never trained for racing (canter and gallop) before the study. Each horse followed a feeding program to enhance health, performance and well-being. Horses were fed three times a day (8:00 a.m., 12:00 a.m. and 5:00 p.m.) with hay (3 kg/horse day as fed) supplemented with green grass and mixed cereal concentrate (3 kg/horse day as fed). Water was available ad libitum. 

Horses were trained with the same training schedule (Table 1). Training was performed from Monday to Saturday with one-day rest (Sunday) for each horse, for a 120-day period. Horses were tested 30 days before (T-30) the beginning of the training program including canter and gallop (T0) and at 30 days (T30), 60 days (T60) and 90 days (T90) after T0. At the end of the training period, bronchoalveolar lavage (BAL) examinations were performed on each horse to exclude EIPH. Each animal had one or more races after the end of the experimental period. No horses showed poor performance syndrome during the study period or the races. 

### 2.3. Sample Collection

The sampling activity was performed once a month, from March 2018 to July 2018, at 6:30 a.m., at rest, before training and at feeding. The experimental period was divided into five times starting one month before the beginning of the official training period to the end of the experimental period (T-30, T0, T30, T60 and T90). March (T-30) was considered as the month in which the animals started light canter; April (T0) was the first month of training simulating competitions (gallop). From April to July, training was incremental. For each time, blood samples were collected by venipuncture of the jugular vein, paying attention to avoid unnecessary manipulation of the sampling site, which could result in activation of coagulation. Blood samples were collected into vacutainer tubes (Terumo Corporation, BD brand; Tokyo, Japan) with anticoagulant additives (10 mL vacutainer tubes containing K3-ethylenediamine tetra-acetic acid (EDTA) and 3.6 mL vacutainer tubes containing 3.8% sodium citrate). K3-EDTA tubes were used to determine the complete blood count (CBC). Sodium citrate tubes were used to evaluate the clotting parameters (prothrombin time—PT, activated partial prothrombin time—PTT, thrombin clotting time—TCT, fibrinogen) and the complex TAT. All the tubes and samples were immediately transported to the local hospital for same-day analysis and were analyzed within 3 h after collection.

### 2.4. Laboratory Analysis

CBC, including the leukocyte differential counts, was performed using an automated hematology analyzer (Sysmex-XT1800iV; Sysmex, Kobe, Japan) validated for horses and equipped with multispecies software [29,30]. This analyzer combines laser-based flow cytometry and impedance technology. Quality control and calibration were weekly performed with the e-check Xe (Sysmex). The following analytes were measured: red blood cells (RBC), hemoglobin (Hgb), hematocrit (Hct), mean corpuscular volume (MCV), mean corpuscular hemoglobin (MCH), mean corpuscular hemoglobin concentration (MCHC), platelets (PLT), white blood cells (WBC), neutrophils, lymphocytes, monocytes, eosinophils and basophils. Two blood smears for each blood sample were performed and microscopically examined for platelet clumps and manual platelet count was performed for each time point.

#### 2.4.1. Clotting Parameters

Prothrombin time (PT), activated partial thromboplastin time (APTT), thrombin clotting time (TCT) and fibrinogen concentration were assessed on citrated plasma by a standard kit made especially for the ACL TOP coagulometer (Werfen, Bedford, MA, USA) [29,31]. 

#### 2.4.2. TAT Complex 

The TAT complex plasma concentrations were measured by ELISA (Enzygnost TAT kit, Siemens Healthcare Diagnostic, Marburg, Germany. Spectrometer brand: Ortho Clinical Diagnostics (New Jersey, USA)), according to the manufacturer’s instructions [28]. 

### 2.5. Statistical Analysis

Raw data from the CBC were imported into R (v. 3.4.1) (R Core Team (2020). A language and environment for statistical computing. R Foundation for Statistical Computing, Vienna, Austria. URL https://www.R-project.org/ (accessed on 18 October 2020). ver. 3.4.1) for statistical analysis. The NLME library [32] was used to implement a linear multilevel mixed model, setting the animals as a random effect. Descriptive statistics parameters were estimated using the psych library [33]. The differences between time points were analyzed by ANOVA. Dunnett’s post hoc test was used to compare the mean of all groups to that of the T-30 group (control group) using the “emmeans” R package (Russell Lenth (2020). emmeans: Estimated Marginal Means, aka Least-Squares Means. R package version 1.5.2-1. https://CRAN.R-project.org/package=emmeans (accessed on 18 October 2020), setting the significance at *p* < 0.05. All results are expressed as mean ± standard deviation (SD). For clotting parameters and TAT, differences among the time points were analyzed by Kruskal–Wallis one-way analysis of variance, Dunn’s multiple comparison post-test and Fisher’s exact test and mean ± SD was calculated (Graph Pad Statistics software). A *p* < 0.05 level was considered statistically significant. The correlation between the variation in the eosinophil content and the platelets, fibrinogen, PT, APTT and TCT among the sampling times was performed by applying the rcorr function of the “Hmisc” R package by calculating Pearson’s coefficient and the relative statistical significance.

## 3. Results

### 3.1. Complete Blood Count

**Figure 1:**
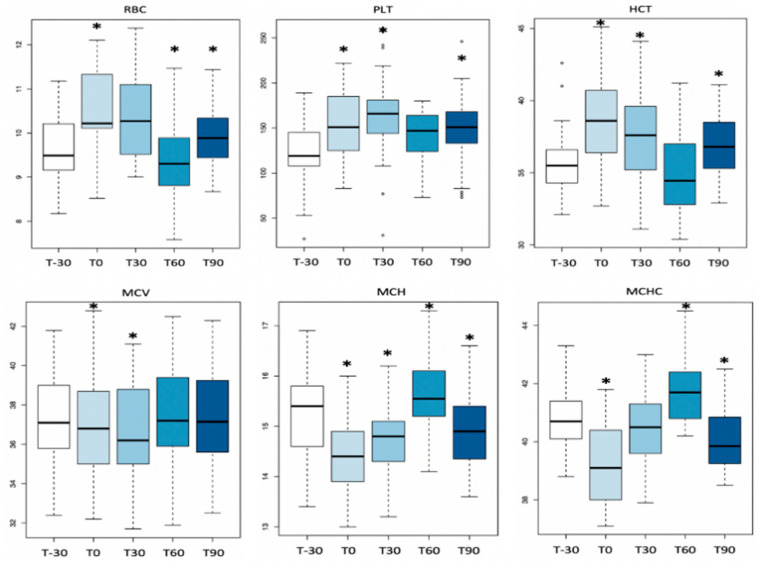

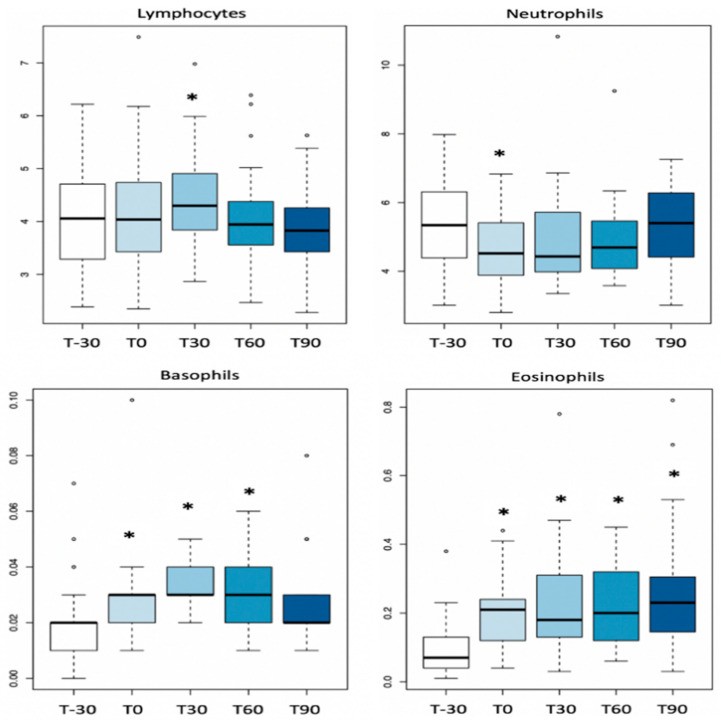
Boxplot with significant CBC parameters in the time series. Circles in the plot area represent outliers. Asterisks (*) in the plot area represent time points that statistically differ from T-30.

Table 2 shows mean levels ± standard deviation (SD) of CBC parameters for each time point and the statistical differences during the training period (*p* < 0.01). A significant effect of training was observed compared with the beginning of the activity (T-30) for RBC (T0, T30, T90), HCT (T0, T30, T90), MCV (T0, T30), MCH (T0, T30, T60, T90), MCHC (T0, T60, T90), PLT (T0, T30, T90), RDW (T30), neutrophils count (T0), lymphocyte count (T30), eosinophils count (T0, T30, T60, T90) and basophils count (T0, T30, T60). RBC, HCT, PLT and lymphocyte, eosinophils and basophils counts increased, whereas MCV, MCH, MCHC, RDW and neutrophils count decreased (Figure 1). Mean values, with the exception of MCV, MCH and lymphocytes, were within the reference ranges. Manual platelet counts were similar to automated platelet counts and no differences were identified. Although there was an r coefficient of 0.65 between eosinophils and platelets, this was not statistically significant (*p* = 0.2).

### 3.2. Clotting Parameters

**Figure 2:**
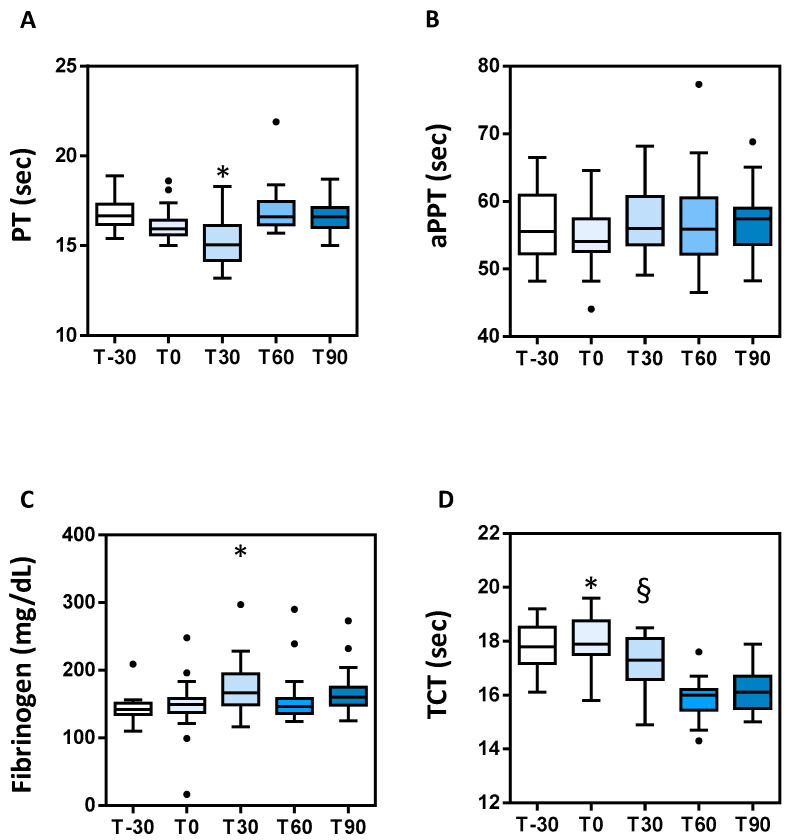
Boxplot with coagulation parameters in the time series. (**A**) Prothrombin time (PT), * = *p* < 0.05 vs. all; (**B**) activated partial thromboplastin time (APTT); (**C**) fibrinogen * = *p* < 0.05 vs. T-30 and T60; (**D**) thrombin clotting time (TCT) * = *p* < 0.05 vs. T30, T60 and T90, § = *p* < 0.05 vs. T60 and T90. Circles in the plot area represent outliers.

The clotting parameters changed during the experimental time, providing a picture of hypercoagulability at T30, with the exception of APTT. In fact, fibrinogen levels were increased, while PT and TT were shorter. Most of these parameters tended to return to the baseline value at T90 (Table 3, Figure 2). However, mean values remained within the reference ranges.

### 3.3. TAT Complex

**Figure 3:**
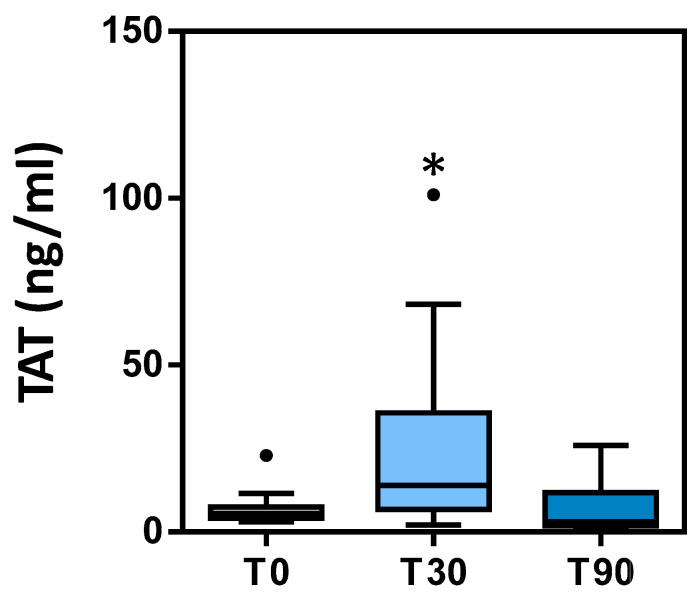
TAT levels measured at T0, T30 and T90. Circles in the plot area represent outliers. * *p* < 0.05 vs. T0.

Plasma TAT complex levels increased at T30 compared to T0 and T90, where they tended to return to basal levels (Table 3, Figure 3). Mean values were within the reference ranges at T0 and T90; however, at T30, they were at the upper limit of the reference range.

## 4. Discussion

The results of the current study show that the first long-term training period in previously never trained Thoroughbreds induces significant changes in CBC values, clotting parameters and TAT levels, mostly evident after 30 days of training.

The analyzed hematological parameters changed in response to training in a substantially similar way to what has been previously described in humans and horses [38,39]. A significant increase in red blood cell indexes (higher HCT and RBCs count) with reduced red blood cell volume (lower MCV) and decreased hemoglobin content (lower MCH and MCHC) was detected during training (T0, T30 and T90 time points) compared to before training levels (T-30). Long-term training was previously shown to increase circulating red blood cell mass due to the release of erythrocytes into the bloodstream for enhancing oxygen transport [1]. In training racehorses, hematological and cardiovascular adaptations are needed to guarantee the required oxygen transport and blood-borne substrates to muscles as well as to remove metabolites, and they have been recognized as factors correlated with the level of training and effort [1]. Further, a decrease in plasma volume has been demonstrated to contribute to the circulating increase in red cell parameters in elite athletes following high-intensity interval training [21]. However, the relationship of smaller RBC volumes with physical fitness was previously unknown in horses. Decreased mean corpuscular volume (MCV) at T0 and T30, with mean values slightly below the reference range, and reduced hemoglobin content (MCH and MCHC) of RBC might be related to an early-stage iron deficiency, prior to the development of an anemic state [38]. In human athletes, non-anemic microcytosis has been demonstrated and suggested to be associated with lower anaerobic fitness, but without affecting aerobic fitness in the course of short-to-medium-distance running [38].

As demonstrated in humans [40,41] and in a previous study in Thoroughbreds [7], our results evidence that the first long-term training in previously untrained racehorses induces an increase in PLT count at each time point compared to T-30. Several studies showed that acute exercise causes a transient increase in platelet count [2]. This increase is caused by hemoconcentration and by platelet release mainly from the spleen but also from the liver and lungs [42]. The permanence of these modifications during the first workout season probably indicates activation of platelets due to exercise training. In humans, exercise-induced thrombocytosis has been demonstrated in sedentary people [41] and during high-intensity interval exercise [21]. Considering that platelets play a major role in blood clotting activation, it is conceivable that the exercise triggers hypercoagulability also through platelet activation. Additional data regarding changes in platelet function and aggregability during long-term training have been evaluated in our study and they will be presented in a future report.

As shown in a previous similar study [3], we did not find significant changes in total WBC count, even if there were significant changes in the differential count, with decreased neutrophil count (T0) and significantly increased lymphocyte (T30) and basophil counts (T0, T30, T60), which tended to return to basal levels at T90. Interestingly, we show, for the first time, a progressive increase in the eosinophil count (T0, T30, T60, T90) from the beginning to the end of the training period. In our study, all included horses were clinically healthy and dewormed during the entire research period, and other possible causes of eosinophilia were excluded such as equine asthma. Leukocyte and differential counts were within the reference ranges, with the exception of lymphocytes showing a mean value at T30 slightly higher than the upper reference interval. Lymphocytosis may be caused by subclinical viral infection or due to the increase in IL-8 expression. Leukocytosis, mostly due to an increase in neutrophils, can also be caused by excitation (adrenaline) connected with blood sampling.

Literature data suggest that an increased leukocytes count is among the most consistent responses to exercise, generally occurring after all types of exercise. Exercise-induced leukocytosis is often compared with an inflammation-like reaction and the increase is quantitatively related to the response induced by physiological insults to the immune system [3,43]. Exercise-induced leukocytosis is also considered as pseudoleukocytosis because it does not seem to be connected with the production of new cells but rather due to the increase in the number of lymphocytes as a result of increased adrenaline secretion consequent to their release in blood from the spleen and, to a minor degree, from the bone marrow and lymph nodes [39].

Recently, it has been reported both in a murine model and in horses that exercise training can promote the immune response by a proliferation of peripheral lymphocytes [43,44,45]. In humans and horses, it was previously observed that interleukin IL-8 increases in the blood of athletes performing exercise and this might explain the increased lymphocytes counts [43,44,45,46].

On the other hand, the increase in eosinophil granulocytes may be related to intense exercise-induced physical effort and stress during training [1]. Increased levels of the chemokine eotaxin/CCL11 detected in sportsmen, a potent chemoattractant and activator of human eosinophils, may be responsible for the recruitment of eosinophils into the circulation in racehorses [45]. Until recently, eosinophils were considered as a major force in parasite defense and as mediators of allergic disease, but an increasing amount of data suggest also a homeostatic role of these cells during the resolution of inflammation and in tissue repair [47]. In addition, in humans, strenuous exercise has been proven to promote eosinophil–platelet aggregation induced by oxidative stress and inflammatory mediators. Indeed, several cytokines enhance platelet reactivity and increase the capacity of platelets to adhere to leukocytes. In particular, vigorous exercise increases the amount of *IL1B* released from muscles and produced by lymphocytes [46], and this may promote the reactivity of platelets and their capacity to adhere to leukocytes by modulating the expression of adhesion molecules on platelets and leukocytes [47,48]. Such heterotypic adhesive interactions seem to increase inflammatory responses and accelerate thrombus formation in the microcirculation [49,50,51,52].

Interestingly, in our study, we detected an increase in both platelet and eosinophil counts, in addition to enhanced *IL1B* expression (data reported in our previous study [4,43], during the entire training period. These parameters could be closely interrelated since, at least from the functional point of view, the two cells have been proven to strictly cooperate [3,43]. In this regard, eosinophils have recently been reported to accumulate in human thrombi [53] and to express tissue factor, a strong trigger of blood coagulation [54]. Moreover, activated eosinophils were shown to bind to immobilized platelets under blood flow in the yet unstable thrombi [55]. Recent data show that hypereosinophilic human patients indeed suffer from an increased incidence of atypical thrombotic events [56]. Evidence supporting a direct contribution of eosinophils to the hemostatic process has recently been obtained in humans and in a murine model [3,43]. In particular, it has been shown that eosinophils are able to autonomously generate thrombin by expressing tissue factor and 12/15-lipoxygenase (12/15-LO), a key enzyme promoting the generation of procoagulant phospholipids at their surface [57]. Indeed, eosinophils have been found to accumulate at the border of platelet-rich areas of thrombi formed on vascular injury in mice, where they seem to contribute to thrombin formation and thrombus stabilization. Conversely, in eosinophil-deficient mice, a significantly decreased thrombotic potential was detected without alterations in the platelet number and function of clotting factors. These findings show eosinophil-induced secondary hemostasis and confirm that platelets and eosinophils play a synergistic role for thrombosis. The conservation of this enzymatic pathway during mammalian evolution suggests a key role of eosinophils in exercise injury-induced thrombin generation and blood coagulation also in training racehorses.

For the above-mentioned reasons, we assessed, for the first time, the correlation of eosinophil and platelet counts with clotting factors alterations in horses undergoing long-term training. We found positive correlations between eosinophils and platelets (r = 0.64), and eosinophils and fibrinogen levels (r = 0.81), as well as a strong negative correlation between eosinophils and TCT (−0.71), even if not to the levels of statistical significance (*p* = 0.2; *p* = 0.09 and *p* = 0.17, respectively), possibly due to the inter-individual variability between horses. Therefore, additional studies are required to fully investigate these correlations.

It is worth mentioning that activated platelets, able to bind eosinophils, play an important role in the pathogenesis of allergic asthma and in chronic obstructive pulmonary disease—equine asthma and exercise-induced pulmonary hemorrhage (EIPH)—in racehorses [49,50,58,59]. Our data suggest that future investigations should be conducted in horses with equine asthma and EIPH to clarify if the interaction between platelets and eosinophils can have a key role in the hypercoagulability generated by training and thus to identify novel targets for the prevention and treatment of exercise-induced thrombotic disease.

There are few data on the effect of long-term acute exercise training on the hemostatic system [46]. The present study demonstrates that the clotting system shows increased activity (decreased PT and TCT times, increased fibrinogen concentrations) already one month after the start of training and tends to return to basal levels 3 months later. Even if their mean values fell within the physiologic range of horses, these data suggest a pro-coagulative status. These results are in agreement with data obtained in humans [12] and in Thoroughbreds [3,7]. Nevertheless, differently from previous studies in Thoroughbreds, we found that the greatest variations in clotting parameters [7] during long-term training were reached at T30. Interestingly, we show that this adaptive response occurs earlier compared with what has previously been reported (T30 vs. T60) [7]. Moreover, we demonstrate that after 3 months of training, the horses had adapted to the exercise program since clotting parameters returned to baseline.

Our results differ from those previously reported in other types of efforts such as those by Piccione et al. [10], who did not find significant alterations in clotting parameters during 10 weeks of a high-level show jumping training program in adult trained Standardbred and Italian Saddle Horses, although this study had some limitations, such as the inclusion of few horses, the majority of which were elderly and gelding (hormones may influence the coagulative status and response to training). Moreover, the studied horses had probably adapted to training and possibly prolonged and more intense exercise would have been required to trigger hypercoagulability. These results suggest that monitoring of hemocoagulation during training programs in racehorses may be useful to assess the fitness of animals and to adjust training schedules.

Hypercoagulability at T30 compared to pre-training was confirmed also by the significantly increased levels of the plasma TAT complex. TAT is a molecular complex of thrombin and antithrombin (AT), a primary thrombin inhibitor, in a 1:1 ratio [58]. Moreover, the return of TAT to basal levels at T90 confirms the adaptation to exercise. TAT indicates in vivo thrombin generation and serves as a marker of prothrombotic status. Thrombin levels cannot be measured directly due to its short half-life in blood, whereas the TAT complex is directly measurable, having a half-life of about 10 min [25]. Consequently, TAT is used to assess in vivo thrombin formation and its increase indicates a prothrombotic status detected earlier than by other laboratory assays. TAT has been found to be increased in humans with DIC, deep vein thrombosis and pulmonary thromboembolism [60]. Some studies in humans have shown increased TAT levels after physical exercise [61,62,63]. In veterinary medicine, there are very few studies on the use of TAT. In horses, it has been considered a marker for hypercoagulation in colic, laminitis and EHV-1 infection, as well as during endurance exercise [64]. To our knowledge, this is the first report showing increased TAT complexes in Thoroughbreds during the first long-training program. In general, we detected values higher than those found in other studies.

## 5. Conclusions

In young untrained Thoroughbreds, the first long-term training period was found to induce changes in some hematological and clotting parameters with increased red blood cell indexes, increased lymphocytes, eosinophils and platelet counts, increased fibrinogen and TAT levels and shorter PT and TCT times. The observed modifications on platelet count and clotting parameters suggest the development of a hypercoagulative condition due to exercise training.

In young Thoroughbreds during the first workout season, it seems that most of the parameters reached the maximum levels of changes after 1 month of training and that the adaptive response to conditioning began after two months of training and reached the basal levels 3 months from the beginning of the season, suggesting that horses had adapted to the training program. More studies are needed to deeply investigate the functional role of platelets and eosinophils in these coagulative changes in the training racehorse and to test the use of TAT as an early marker of prothrombotic status in these animals.

## Figures and Tables

**Table 1:** Table showing the daily training program completed by each horse involved in the study. Speeds: Walk: 100 m/min, Trot: 200 m/min, Canter: 350 m/min, and Gallop: 1000 m/min (min: minutes).

March (T-30)	April (T0)	May (T30)	June (T60)	July (T90)
15 min Walk	15 min Walk	15 min Walk	15 min Walk	15 min Walk
10 min Trot	10 min Trot	10 min Trot	10 min Trot	10 min Trot
Rest	6 min Canter	6 min Canter	6 min Canter	6 min Canter
10 min Trot	Tuesday:	Tuesday:	Tuesday:	Tuesday:
Walk	1 min Gallop	2 min Gallop	3 min Gallop	4 min Gallop

**Table 2:** Means ± SD of completed blood count (CBC) parameters. Parameters significantly modified compared with T-30 are indicated in bold.

Parameters	T-30	T0	T30	T60	T90	RIs of 2-Year-Old Thoroughbred Horses in Training [29,31,34]
**RBC** **(**×10^12^/L**)**	9.6 ± 0.7	**10.4 ± 0.9**	**10.3 ± 0.9**	**9.4 ± 0.8**	**9.9 ± 0.70**	8.7–11.7
**Hgb** **(g/dL)**	14.5 ± 1.1	15.0 ± 1.3	15.1 ± 1.3	14.6 ± 1.2	14.3 ± 2.0	12.8–16.6
**Hct** **(%)**	35 ± 2.0	**38 ± 3.0**	**37 ± 3.0**	35 ± 3.0	**36 ± 2.0**	34–45
**MCV** **(fL)**	37.2 ± 2.4	**36.8 ± 2.6**	**36.3 ± 2.5**	37.4 ± 2.5	37.0 ± 2.5	37.0–42.1
**MCH** **(pg)**	15.1 ± 0.8	**14.4 ± 0.7**	**14.7 ± 0.7**	**15.5 ± 0.8**	**14.8 ± 0.7**	13.7–15.7
**MCHC** **(mg/dL)**	40.7 ± 1.1	**39.2 ± 1.3**	40.5 ± 1.3	**41.7 ± 1.0**	**40.1 ± 1.1**	35.9–37.9
**PLT** **(**×10^9^/L**)**	123 ± 37	**151 ± 35**	**166 ± 52**	142 ± 25	**149 ± 42**	127–206
**WBC** **(**×10^9^/L**)**	9.9 ± 1.9	9.5 ± 1.6	10.2 ± 1.9	9.7 ± 1.5	9.9 ± 1.5	7.3–12.7
**Neutrophils (**×10^9^/L**)**	5.3± 1.3	**4.7 ± 1.1**	5.0± 1.6	4.9 ± 1.2	5.2 ± 1.2	4.0–6.0
**Lymphocyte (**×10^9^/L**)**	4.01 ± 0.92	4.16 ± 1.09	**4.42 ± 0.97**	4.06 ± 1.03	3.90 ± 0.78	2.7–4.4
**Monocytes (**×10^9^/L**)**	0.45 ± 0.10	0.46 ± 0.11	0.49 ± 0.10	0.49 ± 0.12	0.49 ± 0.09	0.26–0.56
**Eosinophyls (**×10^9^/L**)**	0.09 ± 0.07	**0.19 ± 0.09**	**0.22 ± 0.16**	**0.22 ± 0.11**	**0.27 ± 0.18**	0–0.3
**Basophyls (**×10^9^/L**)**	0.02 ± 0.01	**0.03** **± 0.01**	**0.03** **± 0.01**	**0.03** **± 0.01**	0.02 ± 0.01	0–0.2

RBC: red blood cells; Hgb: hemoglobin; Hct: hematocrit; MCV: mean corpuscular volume; MCH: mean corpuscular hemoglobin; MCHC: mean corpuscular hemoglobin concentration; Plt: platelets; RDW: red blood cells distribution width; WBC: white blood cells; Neut: segmented neutrophils; Lymph: lymphocytes; Mono: monocytes; Eo: eosinophils; Baso: basophils; RIs: reference intervals.

**Table 3:** Means ± SD of clotting parameters. Parameters significantly modified are in bold.

Parameters	T-30	T0	T30	T60	T90	Ris [34,35,36,37]
**PT (** **s** **)**	16.8 ± 0.16	16.1 ± 0.14	**15.26 ± 0.27 ^a^**	16.91 ± 0.25	16.63 ± 0.18	10–17
**APTT (** **s** **)**	56.76 ± 0.90	54.97 ± 0.79	56.80 ± 0.91	56.84 ± 1.29	56.73 ± 1.09	27–58
**TCT (** **s** **)**	17.83 ± 0.14	**18.08 ± 0.16 ^b^**	**17.26 ± 0.17 ^c^**	15.86 ± 0.13	16.13 ± 0.14	15–19
**Fbg (mg/dL)**	142 ± 3.1	148.8 ± 6.5	**172.8 ± 7.5 ^d^**	155 ± 6.9	166.9 ± 6.4	100–400
**TAT (ng/mL)**	-------	6.33 ± 0.5	**23.44 ± 3.7 ^e^**	-------	7.03 ± 1.6	1.95–9.03

PT: prothrombin time, APTT: activated partial prothrombin time, TCT: thrombin time, Fbg: fibrinogen; TAT: thrombin–antithrombin complex; RIs: reference intervals. ^a^ = *p* < 0.05 vs. T-30, T0, T60, T90. ^b^ = *p* < 0.05 vs. T30, T60, T90. ^c^ = *p* < 0.05 vs. T60, T90. ^d^ = *p* < 0.05 vs. T-30, T60. ^e^ = *p* < 0.05 vs. T0, T90.
